# The Role of Intermetallic Compounds in Controlling the Microstructural, Physical and Mechanical Properties of Cu-[Sn-Ag-Cu-Bi]-Cu Solder Joints

**DOI:** 10.1038/s41598-019-44758-3

**Published:** 2019-06-10

**Authors:** Reza Sayyadi, Homam Naffakh-Moosavy

**Affiliations:** Department of Materials Engineering, Tarbiat Modares University, Tehran, Iran

**Keywords:** Aerospace engineering, Electrical and electronic engineering

## Abstract

A lead-free Sn-2.5Ag-0.7Cu base solder with different weight percentages of bismuth (0, 1, 2.5, 5) was used. Thermal properties, microstructure, wettability and mechanical properties were investigated. By decreasing the degree of undercooling, microstructure improved, the eutectic structure become finer and the size of β-Sn and intermetallic compounds decreased. By the addition of bismuth to SAC257 solder, the spreading ratio increased from 80.46% to 85.97, indicating an improvement in wettability. In order to investigate the joint properties, alloy solders were bonded to copper substrate, and the structure of the interface, tensile-shear strength and the fractured surfaces were studied. It was observed that the thickness of the intermetallic compounds of Cu_6_Sn_5_ at the interface decreased with the addition of bismuth, and the lowest thickness of the interfacial IMCs was found in the SAC257-1Bi solder joint, which decreased about 14% compared to the base solder. Also, the Cu/SAC257-1Bi/Cu bond had the highest tensile-shear strength and elongation percentage among the alloy solders, which has a tensile-shear strength of about 30% and an elongation percentage of about 38% higher than the base solder joint.

## Introduction

In recent years, the use of lead-free solders has increased to replace with the common Sn-Pb solder^1–3^. Among the various groups of lead-free solders, the Sn-Ag-Cu (SAC) triple alloy has the highest acceptability^4^. Due to this fact that these solders are easy to use and have relatively suitable mechanical and physical properties, they are the most widely used alloy solders^5–8^; However, there are still many unresolved issues with these solders^9,10^. SAC solders with high Ag content (>3 wt.%) have good cyclic thermal properties. However, the formation of a large amount of Ag_3_Sn intermetallic compound (IMC) results in non-homogeneous stress distribution and reduction of reliability in these solders^11,12^. Also, the high silver content has decreased the wettability and has increased the price of these solders. Recently, the SAC solder problems with high percentage of silver have been solved by SAC solders containing low silver content (1–3 wt.%). By reducing the percentage of Ag, despite an increase in elastic compliance and the plastic energy dissipation ability, thermal properties such as thermal fatigue and creep life of SAC solders have been reduced^11,13^. Therefore, to improve the properties of these solders, series of investigations have been carried out which show that the properties of SAC solders can be improved by adding a small amount of alloying elements such as Ni, Ti, Sb, Bi, Zn, Ni, Ga. Adding micro-alloying elements can modify the microstructure of SAC solders and it can limit the formation of Ag_3_Sn intermetallic by affecting the solidification rate^11,14,15^.

Zhang *et al*.^16^ studied the effect of Zn on the wettability of Sn-3.8Ag-0.7Cu solder. They have shown an increase in the wettability of the mentioned solder by adding up to 0.8 wt.% Zn, but decreasing at higher percentages. This is due to the oxidation of Zn during soldering that reduces wettability^16^. Sungkhaphaitoon and Plookphol^17^ studied the effect of Sb addition on microstructure, mechanical and thermal properties of Sn-3Ag-0.5Cu solder. They reported that with the addition of Sb to the mentioned solder, more homogeneous distribution of IMCs was obtained, and the nature of IMCs changed to Ag_3_(Sn,Sb) and Cu_6_(Sn,Sb)_5_ compounds. Moreover, tensile strength increased and the ductility of the solder was reduced. It is worth to mention that by increasing the concentration of Sb from 0 to 3 wt.%, the melting temperature slightly increased^17^. El-Daly *et al*.^9,10^ showed that with the addition of bismuth to the SAC157 solder, the eutectic temperature decreases and the microstructure improves, which is a cause of decrease in the degree of undercooling. In addition, the tensile and creep properties of bismuth-containing solders improve as well^9,10^.

Adding alloying elements to the SAC solders have three major effects on the interface reactions and the formation of IMCs^18^:

- It can increase or decrease the rate of reaction/growth.
- It can change the physical properties of the formed phases.
- It can create additional reactive layers at the interface or even replace reactive products with commonly formed IMCs.

These elements generally fall into two general categories: (a) Elements that only affect the activity of the elements participating in the interactions of the interface, and these elements do not affect the formation of these phases. These elements generally have no significant solubility in the IMC layer, such as iron and silver. (B) Elements that participate in the interface interactions and have broad solubility in IMCs, such as gold and nickel. T. Laurila *et al*.^18^ showed that the elements of group (B) have a small effect on the growth of IMCs, while the elements of group (b) have a significant effect. The results showed that the addition of silver and iron, only reduced the diffusion coefficient, while in the case of gold and nickel, the reaction layer changed to (Cu, Au)_6_Sn_5_ and (Cu, Ni)_6_Sn_5_, respectively, and even in the case of nickel, the curvature of IMC growth did not show any other parabolic behavior with time, indicating a change in the growth mechanism^18^. Tao *et al*.^19^ added Ni and Sb to the Sn-3.8Ag-0.7Cu-3Bi alloy solder. They showed that shear strength increases with the solid solution mechanism of Sb in Sn matrix, and reinforcement of new intermetallic compounds (Cu, Ni) _6_Sn_5_ and Ag_3_(Sn, Sb)^19^. A. Kantarcıoglu and Y.E. Kalay^20^ have shown that by adding micro alloying elements, Fe (0.01–0.1 wt%) and Al (0.05 wt%) to the eutectic SAC solder, the microstructure of the solder improved, the formation of Ag_3_Sn compounds was limited, and the new FeSn_2_ and Al-Sn-Cu intermetallic compounds were formed in the microstructure. Also, the thickness of the IMCs at the interface was reduced and the shear properties of soldered copper improved^20^. Developments of adding Bi and other common elements in Sn-Ag-Cu alloy are summarized in Table 1.

**Table 1:** Common elements/additives in SAC lead-free solder and their effects^5,9,10,15,16,19,32,49–52^.

Element	Characteristics
Zn	• It increases oxides and corrodes readily.
	• It requires strong fluxes.
	• It generally decreases wettability.
	• It facilitates formation of new IMC (Cu,Zn)_6_Sn_5_ in the reaction layer at the interface.
In	• It is very expensive and scarce.
	• It decreases melting temperature.
	• It improves wettability.
	• It renders high ductility and low strength in the case of high In-containing solder alloys.
	• It accelerates oxidation during melting.
Sb	• It improves mechanical properties.
	• It increases the melting temperature slightly.
	• It slightly reduces thermal and electrical conductivity.
	• It is considered toxic (listed on the EACEM list of “not to be used” substances).
	• It forms new IMC Cu_6_(Sn,Sb)_5_ and Ag_3_(Sn,Sb) in microstructure.
Ni	• It decreases degree of undercooling and refine microstructure.
	• It changes interfacial reaction layer from Cu6Sn5 to (Cu,Ni)_6_Sn_5_ and Inhibits Cu dissolution.
	• It increases melting temperature and melting range.
	• It improves the drop strength of low-Ag SAC alloys on Cu pads.
	• It suppresses the growth of the brittle Cu_3_Sn IMC layer.
Ti	• It decreases degree of undercooling.
	• It forms new Ti_2_Sn_3_ IMC which is hard and stiff.
	• It increases mechanical strength in low weight percentages (1 > wt%).
	• It suppresses void formation and coalescence at the Cu (substrate)/Cu_3_Sn interface.
Bi	• It reduces solder cost.
	• It decreases melting temperature.
	• It increases tensile strength.
	• It improves creep and fatigue properties.
	• It increases brittleness and is prone to thermal fatigue in 4.5 < wt%.
	• It increases the solidification range 3 < wt%.
	• It decreases the degree of undercooling and refines the microstructure.
Nanoparticles	• These can be the reactive nanoparticles (metal), and non-reactive nanoparticles, for example the oxides (silica, TiO_2_, Al_2_O_3_, ZrO_2_) and carbon.
	• These can improve or change the microstructure and properties of solders, such as mechanical properties and wettability.
	• These need to improve the production process for homogeneous distribution in the solder matrix.

In this study, bismuth element was used to improve the properties of SAC solder alloy. Previous studies suggest that a significant reduction in the weight percent of Ag in SAC solders causes problems like an increase in the solidification range. For this reason, Sn-2.5Ag-0.7Cu solder alloy with moderate percentage of Ag was chosen as the base alloy. Therefore, in this study, the physical and mechanical properties of SAC257-xBi solder and their joints were investigated.

## Experimental Procedure

In this study, the pure elements of tin, silver, copper and bismuth (purity 99.99 wt.%) were used for the synthesis of Sn-2.5Ag-0.7Cu-xBi solder alloys (SAC257-xBi, x = 0, 1, 2.5, 5). For this purpose, the pure elements of Sn, Ag, Cu and various Bi percentages were placed in the alumina crucible in an atmospheric furnace at 650 °C. KCl-LiCl (1:1) was used as a flux to prevent molten oxidation and melt vaporization. For homogenization, the melt was kept at this temperature for 1 hour and experienced remelting for three times after the solidification of the alloyed solder. Casting the final melt was carried out in a cylindrical steel mold with a diameter of 4 cm and a height of 1 cm with a cooling rate of 6–8 °C/s. The chemical composition of the alloy solders after solidification is given in Table 2. The as cast samples were rolled and turned into sheets of 1 mm and 300 microns for use at the following stage (bonding). The rolled sheets were annealed in oven at 100 °C for 1 hour.

**Table 2:** Chemical composition of synthesized solder alloys (wt.%).

Alloy	Sn	Ag	Cu	Bi
SAC257	Bal	2.5	0.7	**0**
SAC257-1Bi	Bal	2.5	0.7	**1**
SAC257-2.5Bi	Bal	2.5	0.7	**2.5**
SAC257-5Bi	Bal	2.5	0.7	**5**

### Characterization of the solder alloys

In order to investigate the microstructure of alloyed solders, the distribution of grains and IMCs before and after alloying, the samples were sectioned in 5 mm * 5 mm dimensions and then cold mounted. Then, the specimens were prepared according to the standard methods of metallography. For etching, a solution of ethanol [96Vol%], hydrochloric acid [2Vol%] and nitric acid [2Vol%] was used and the microstructure of the solder alloys was investigated by a scanning electron microscopy (Philips-Xl30- & FEI-Quanta -EDS Element silicon drift) and optical microscopy (Olympus-BX51M). MIP software was used for analyzing the microstructure images.

**Figure 1:**
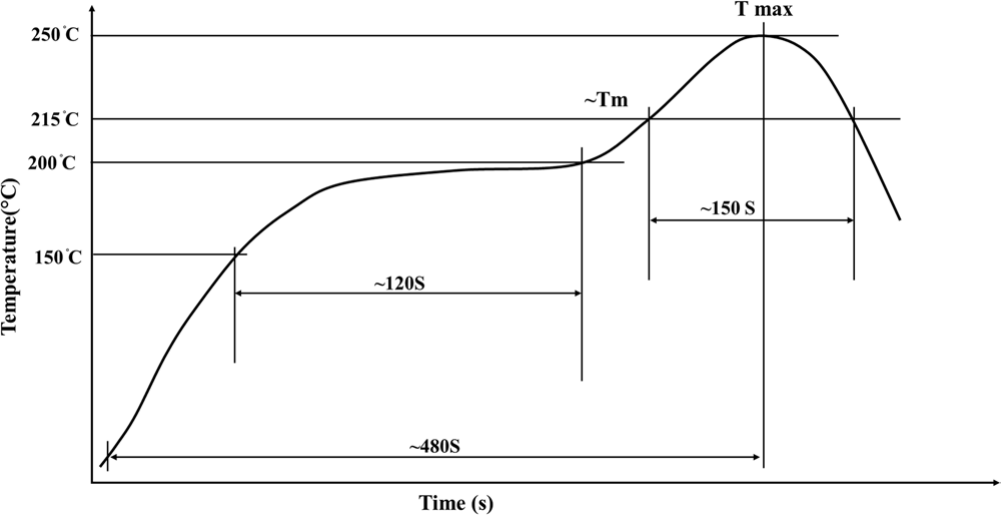
The temperature program to measure the spreading ratio.

**Figure 2:**
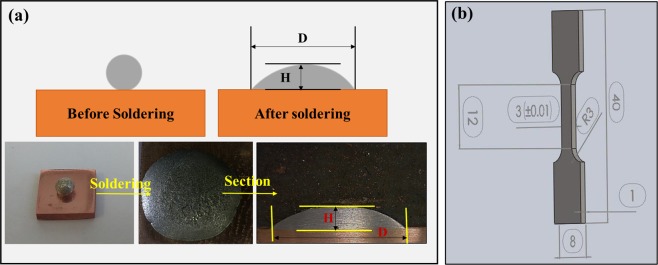
(**a**) The procedure for measuring the spreading ratio, (**b**) the geometry of sub-size samples for tensile test.

The DSC test was used to measure the thermal properties of SAC257-xBi solder alloys (x = 0, 1, 2.5, 5) in accordance with ASTM D3418/E1356 standard by Pishtaz Engineering Co. model TA-1A instrument. Samples with equivalent weight between 15 to 25 mg were used. The test was carried out in the argon atmosphere in the form of sweep (heating-cooling). The heating rate was 10 °C/min and the maximum temperature reached 300 °C during the DSC test. To determine the wettability of the solder alloys according to Japanese Industrial Standard, (JIS 23198-3,2003), the spreading ratio was calculated. For this purpose, the alloyed solder disks of the same weight (200mgr) containing flux were placed inside a ceramic crucible and melted in the furnace to obtain a spherical state after solidification. After the production of spherical shaped solders, they were placed on the copper substrates with flux (RMA) and according to the temperature program represented in Fig. 1; the process was carried out in the furnace. After cooling the specimens, the diameter (D) and height (H) of the solidified droplets were measured and the spreading percentage of the solder alloys was calculated using equation (1). Figure 2a shows how to measure the spreading ratio.

1 $${\rm{Spreading}}\,{\rm{ratio}}=({\rm{D}}-{\rm{H}}/{\rm{D}})\ast 100$$

Where D is the diameter of spread solder (mm), and H is the height of spread (mm).

Tensile test was used to examine the mechanical strength of SAC257-xBi (x = 0, 1, 2.5, 5) solder alloys. For this purpose, the sub-size samples were prepared in accordance with Fig. 2b. The Instron 5500 R test device was used for this test. The test was performed at ambient temperature with a 1 mm/min jaw speed. Three samples were tested for each case and finally the average stress-strain obtained was reported.

### Characterization of the Cu/SAC-xBi/Cu joints

#### Materials

In order to investigate the properties of the solder alloy joints, 2 mm copper sheets with a purity of 99.999 wt.% were used as substrate. For deoxidation and reduction of the surface roughness of copper sheets, the specimens were grinded and washed in sulfuric acid solution for 30 minutes. Finally, in order to ensure the absence of fats and other surface contaminants, the samples were washed with alcohol and kept in the neutral atmosphere of argon until they were bonded to maintain surface conditions. The synthesized solder alloy interlayers were cut in 12* 12 mm^2^ and 300 µm in thickness. In order to remove surface contaminations, the interlayers were washed with alcohol and were stored in the argon atmosphere until bonding process. The RMA flux was used to prevent the base metal and solder alloys against the oxidation during bonding process.

Resistant furnace was used to provide the required temperature for bonding, and bonding temperature program is in accordance with the IEC 60068-2-58 2015 standard, as shown in Fig. 1.

#### Microstructure of the interface

**Figure 3:**
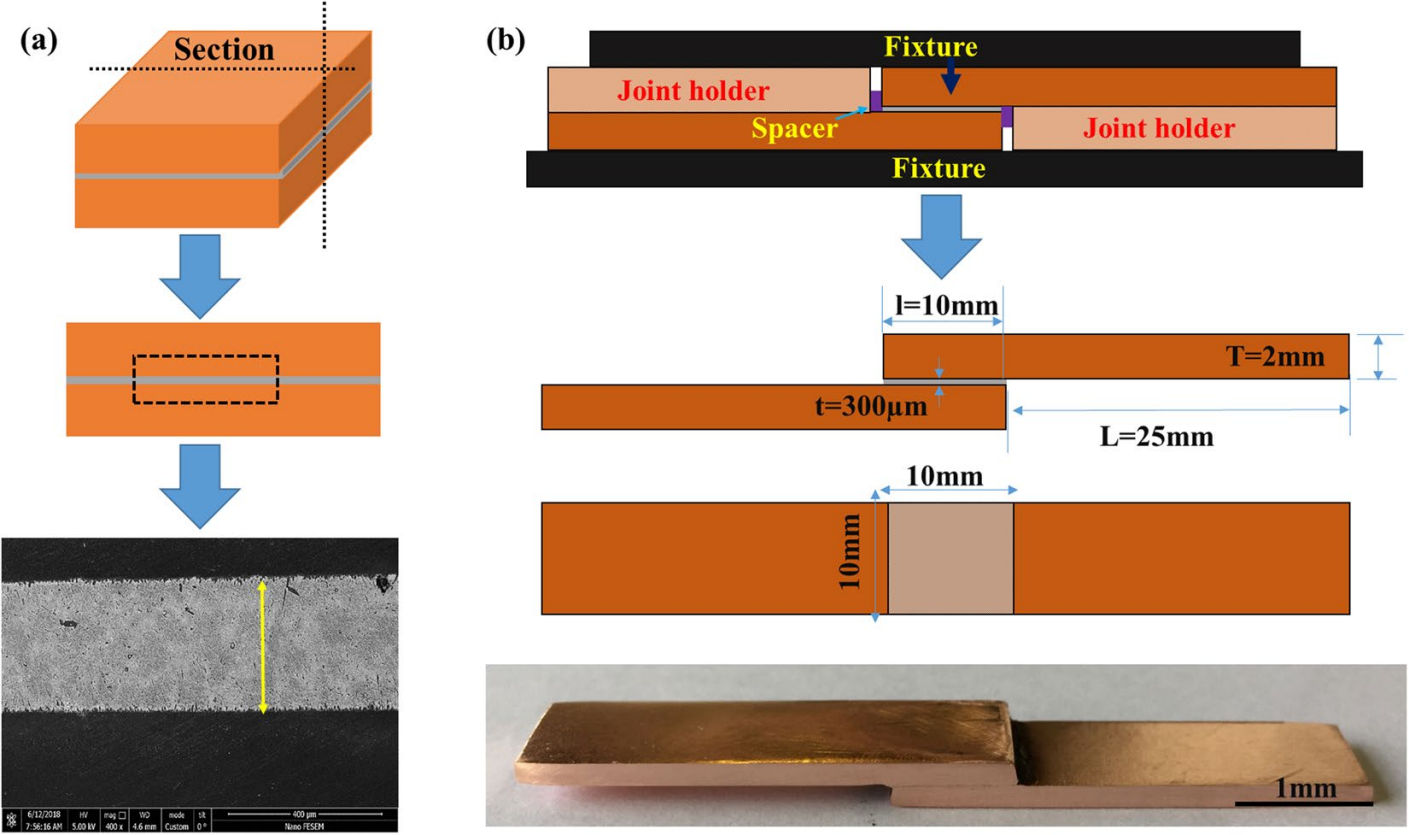
(**a**) The procedure for microstructural characterization of solder joint, (**b**) The details of solder joint set up.

In order to investigate the microstructure of the base solder/ base metal interface, a 300 µm solder foil and flux was placed between two copper samples with dimensions of 10 * 10 * 2 mm, and the system experienced temperature cycle in the furnace in accordance with the temperature program given in Fig. 1. According to the melting temperature of SAC solders, the maximum soldering temperature was chosen at 250 °C. In order to investigate the morphology of the intermetallic compounds formed at the interface after the bonding process was completed, the samples were sectioned perpendicular to the thickness by a cutting machine. The standard metallographic operations were performed on them using a mixed acidic etching solution containing ethanol [96Vol%], hydrochloric acid [2Vol%] and nitric acid [2Vol%]. SEM and FE-SEM were used for microstructural studies (see Fig. 3a). SEM equipped with an EDS system with a silicon drift sensor was used for semi-quantitative analysis of phases. In order to investigate the microstructure, MIP software was used for quantitative calculations of microscopic images such as the size of the interfacial zone and the volume fraction of IMCs and precipitates.

#### Calculation of the IMC thickness

**Figure 4:**
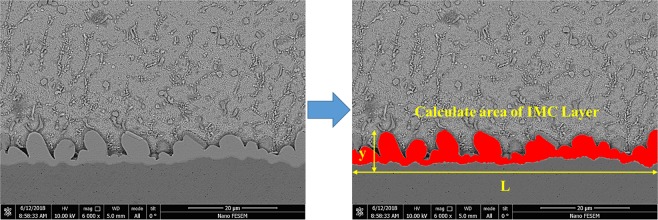
The overall trend of the IMCs thickness calculation by software.

In order to determine the average thickness of the IMCs, the MIP image analyzer software was used. Because the thickness of the IMC layer was not uniform throughout the interface, the mean value of the thickness (Y) of IMC was obtained from the Equation (2), where A is the total area of the IMC layer in the image, L is the length of the IMC in the interface direction. For each of the thicknesses reported in the solder alloys, the length of 400 µm (6 photos) was taken from different parts of the interface region and then calculated and reported. Figure 4 shows the overall trend of the IMCs thickness calculation.

2 $${\rm{Y}}={\rm{A}}/{\rm{L}}$$

#### Tensile-Shear test

The tensile-shear specimens were prepared in accordance with the ASTM D1002 standard which is provided in Fig. 3b. In this test, 300 µm solder alloy foils were used as interlayer, copper samples were used with dimensions of 35 mm * 10 mm * 2 mm, and the soldered area was 10 cm * 10 cm. The temperature program represented in Fig. 1 was used for bonding process. Two pieces of copper with 2 mm thickness were placed on both sides of the sample in order to align the sample in the jaws of the tensile machine. Instron tensile machine was used for tensile-shear test. Test was performed at ambient temperature with 1 mm/min jaw speed. The application of the tensile force with the tensile machine causes a shear force at the interface. Thus, by means of load-displacement diagram, the tensile strength of the joint was measured.

#### Fractography

In order to investigate the failure mechanism of the joint, the tensile-shear fractured samples were sectioned, cleaned in ultrasonic bath for 15 minutes to eliminate the surface contaminations and were investigated by FE-SEM.

## Results and Discussions

### Properties of SAC-xBi solders

#### Thermal and microstructural analysis

**Figure 5:**
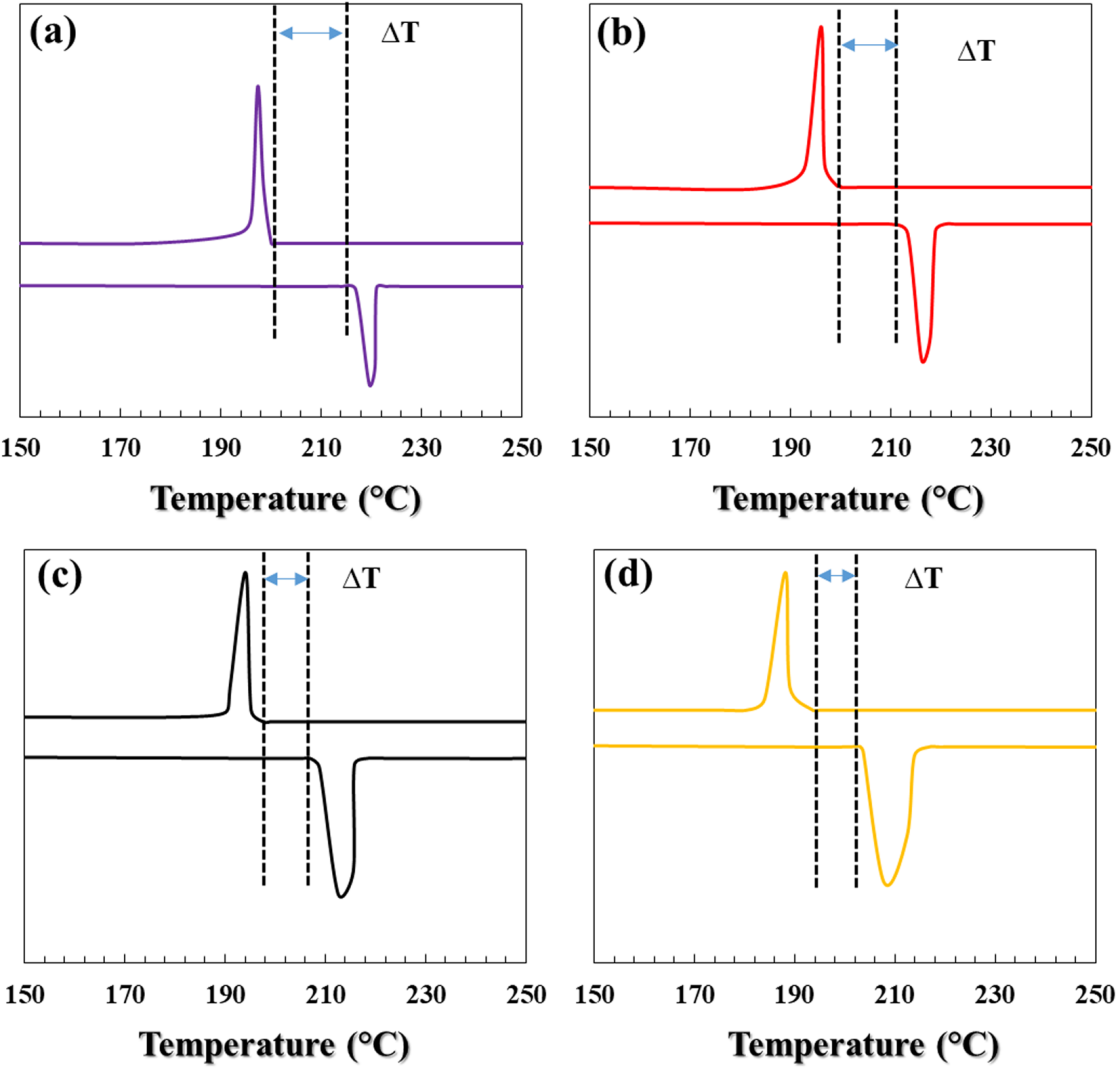
Cooling and heating curves of solder alloys during the DSC test.

**Figure 6:**
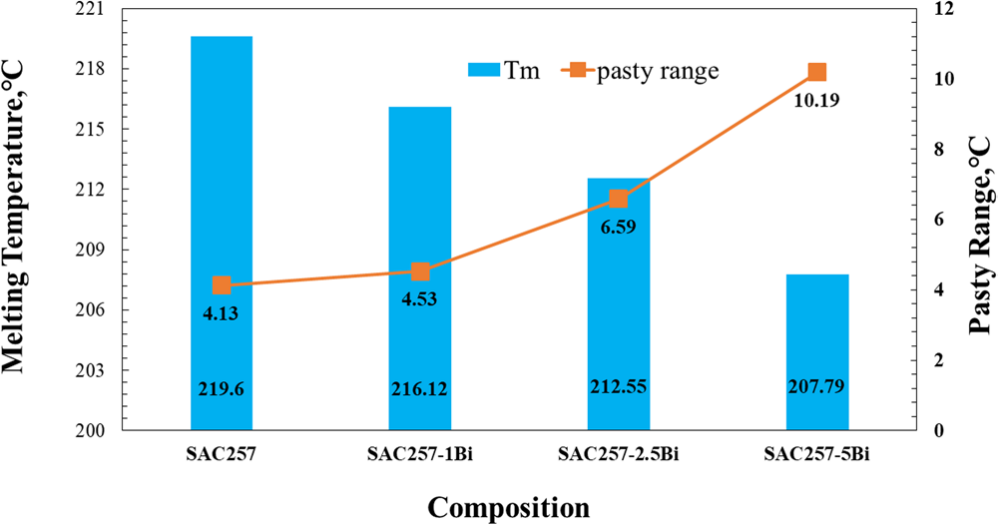
The melting temperature and pasty range of solder alloys obtained from DSC test.

**Figure 7:**
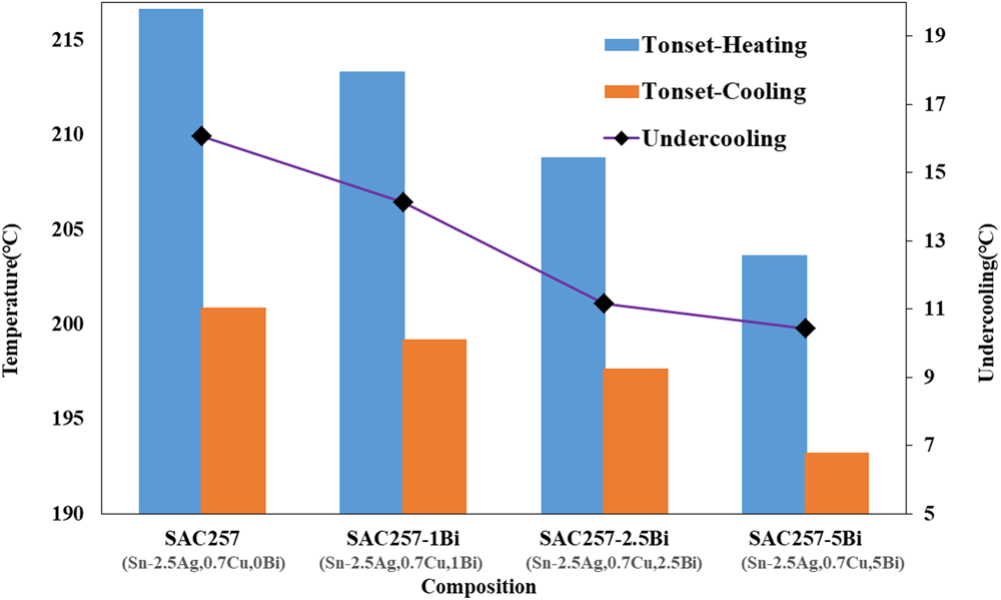
Onset cooling and heating temperatures and undercooling for solder alloys obtained from DSC test.

To study the thermal behavior of solder alloys during cooling and heating, the DSC test was performed. Figures 5, 6 and 7 show the results of this test. The melting temperature is one of the critical factors for solder selection in microelectronic industries, for this reason, this should be taken into consideration to improve the base solder properties^21,22^.

**Figure 8:**
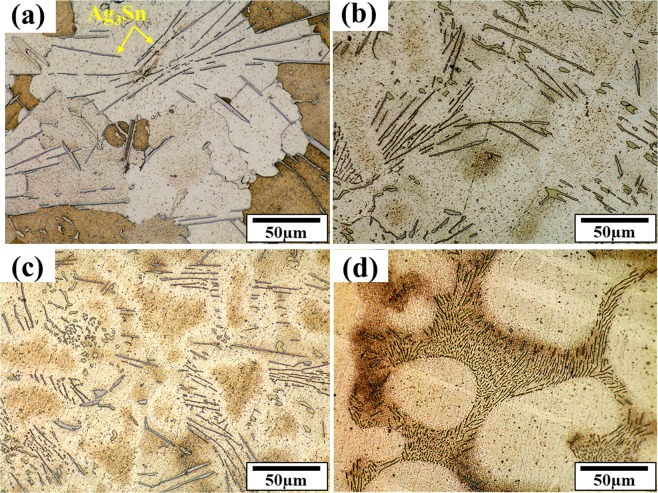
The optical microstructure of solder alloys: (**a**) SAC257, (**b**) SAC257-1Bi, (**c**) SAC257-2.5Bi, and (**d**) SAC257-5Bi.

In this study, with the addition of bismuth to the SAC257 base solder, despite the fact that the change in pasty range is small, the melting point decreases from about 219 °C in the SAC257 base solder to 207 °C in the alloy SAC257-5Bi, which indicates the positive effect of bismuth on the melting point. According to Lindemann’s criterion, melting occurs when the second root of the atom displacement in the network exceeds a certain atomic distance. Therefore, it is possible that with the addition of bismuth to the SAC257 solder, and the formation of new Sn-Bi bonds resulting from the reaction between tin and bismuth, the atomic displacement in crystalline network occurs easier and the overall melting temperature of the system decreases^23–25^. Another parameter affecting the solidification behavior of the solder and its microstructure is undercooling, which is defined as ΔT = T_L_ − T_S_, where T_L_ is the temperature at which the melting starts during heating stage, and T_S_ is the solidification temperature during cooling stage. The lower the ΔT, the better the nucleation of solidified phases, which increases the tendency for heterogeneous nucleation and makes the microstructure finer^9,10,25,26^. According to Fig. 7, the addition of bismuth to the base solder SAC257 has reduced the degree of undercooling, which indicates that the presence of bismuth is very effective in improving the microstructure. As shown in the optical microscopy images in Fig. 8, by adding bismuth to the SAC257 base solder, the eutectic region expands and the β-Sn region is reduced. In SAC257-xBi solder alloys, the decrease in β-Sn size due to the reduction of ΔT can be attributed to the presence of bismuth. Increasing the heterogeneous nucleation sites by primary IMCs, causes faster solidification rate of Ag_3_Sn and β-Sn phases and results in the finer microstructure^9,10,25^.

**Figure 9:**
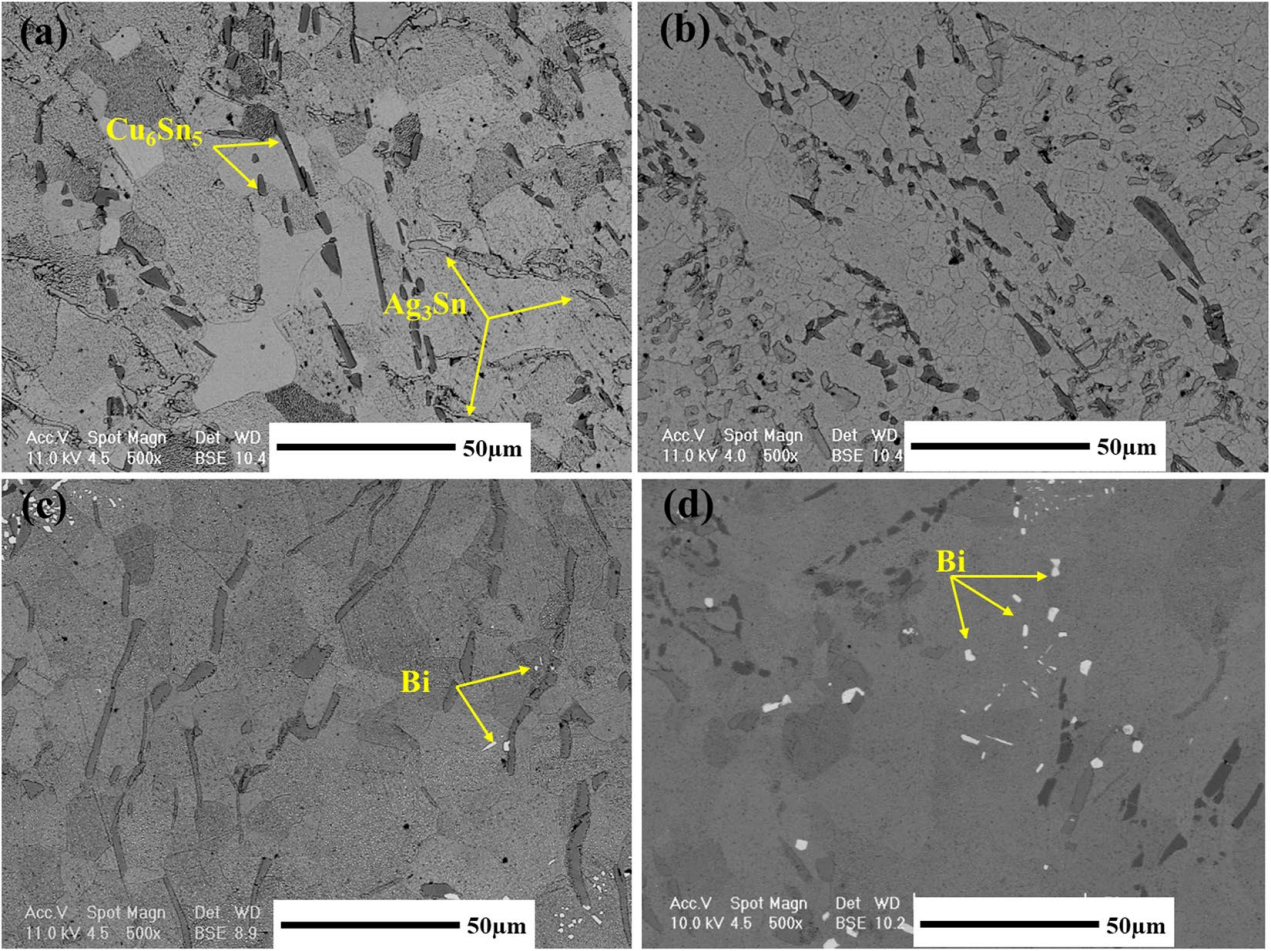
The back-scatter electron SEM images of microstructure for the base solder and SAC257-xBi solder alloys: (**a**) SAC, (**b**) SAC1Bi, (**c**) SAC2.5Bi, and (**d**) SAC5Bi.

**Figure 10:**
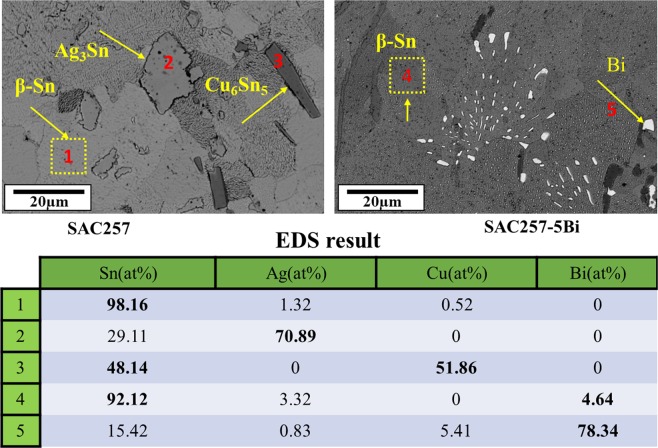
The semi-quantitative EDS analysis of the phases in the base solder and SAC257-5Bi solder alloys.

Figure 9 shows the back-scatter electron SEM images to characterize the microstructure of the base solder and SAC257-xBi solder alloys. According to this fact that the SAC257 base solder chemical composition is close to the eutectic composition, the Ag_3_Sn and Cu_6_Sn_5_ phases are formed in the β-Sn matrix, which are shown in Fig. 9a. By adding bismuth to the SAC257, the microstructure changes so that, according to Fig. 9b–d, the morphology of the Ag_3_Sn and Cu_6_Sn_5_ intermetallic compounds tends to change from needle to equiaxial shape. Also, due to the decrease in the degree of undercooling, the size of the intermetallic compounds decreases^9,10,25^. On the other hand, the Bi solubility is also limited in Sn, and in the case of more than 1 wt.% Bi, bismuth is deposited separately^27,28^, which is visible as white phase in Fig. 9c,d. The semi-quantitative EDS analysis of the phases in the base solder and SAC257-5Bi solder alloys is shown in Fig. 10, which also confirms the presence of white bismuth phase.

**Figure 11:**
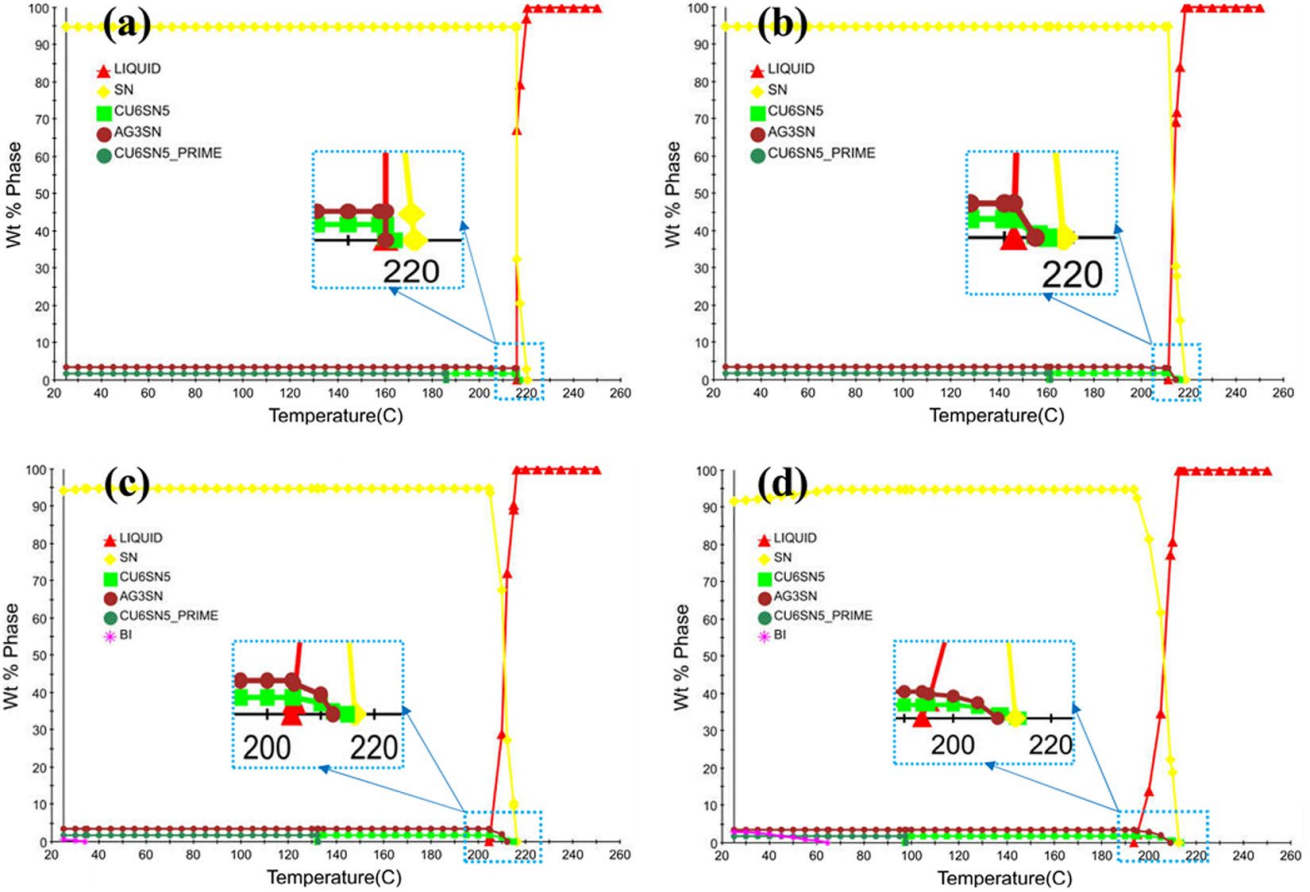
Analysis of the Solidification behavior of solder Alloys: (**a**) SAC 257, (**b**) SAC 257-1 *wt*.*%*Bi, (**c**) SAC 257-2.5 *wt*.*%*Bi, and (**d**) SAC257-5 *wt*.*%*Bi solder alloys by using JMATPRO® software.

**Figure 12:**
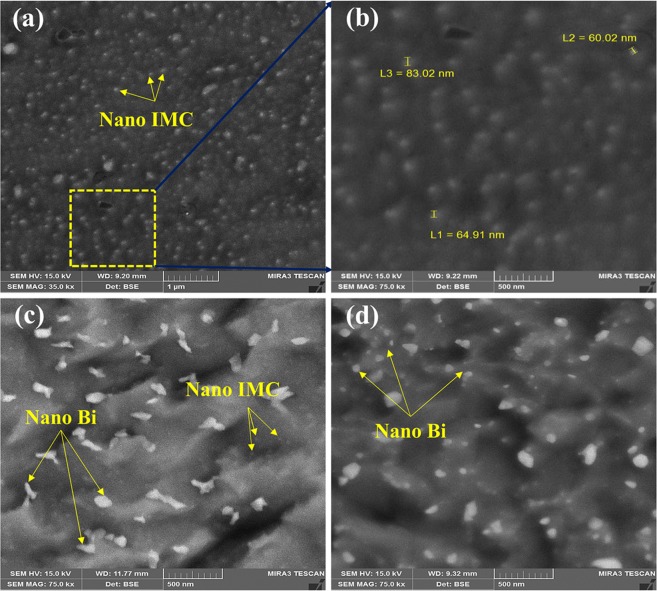
High magnification FE-SEM image from fine IMC and Bi precipitate distribution in SAC 257-Bi Solder alloy: (**a**,**b**) SAC257-1Bi (**c**) SAC257-2.5Bi (**d**) SAC257-5Bi.

Figure 11a–d, represents the results of the solidification sequence through JMATPRO software. As it is evident in SAC257, the intermetallic compounds of Ag_3_Sn and Cu_6_Sn_5_ are formed in the final stages of solidification. But, by adding Bi in the case of SAC257-1Bi, SAC257-2.5Bi, and SAC257-5Bi, the Ag3Sn and Cu6Sn5 intermetallic compounds are formed from the beginning of the solidification process^25^. Studies have shown that the formation of the β-Sn matrix phase in Sn-based alloys requires high degrees of undercooling^11^; therefore, in SAC257 solder with a relatively high degree of undercooling, first, the β-Sn phase is formed as the primary solidification product, and then the intermetallic compounds as the secondary phases. Talking about SAC-Bi solder alloys, Bi affects the solidification behavior of solder and reduces the degree of undercooling, which results in the formation of a portion of the intermetallic compounds as the primary solidification products, which act as the heterogeneous nucleation sites. On the other hand, reduction of undercooling limits the growth of β-Sn, and leads to the finer grain structure. Another part of the intermetallic compounds are formed simultaneously during a Sn-Ag-Cu ternary eutectic reaction. Therefore, two types of morphologies of the intermetallic compounds are expected to be observed in the microstructure of the SAC257-Bi alloys^25^. According to Fig. 12a–d, it can be seen that in the microstructure of Bi-containing solder alloys, in addition to the large IMCs and Bi precipitates, series of fine precipitates are observed in the matrix. For further examination of these fine particles, FE-SEM images were taken at high magnification from these fine precipitates, as shown in Fig. 12a,b. In the microstructure of the SAC257-1Bi solder, according to the Bi solubility limit in Sn and the contrast of the atomic numbers in BSE mode, it is likely that these nanosize particles are the same IMCs. As mentioned before, the reason for the formation of these nano-precipitates is the decrease in the degrees of undercooling, the increase in heterogeneous nucleation and the formation of intermetallic compounds during solidification. In the SAC257-2.5Bi and SAC257-5Bi solders (Fig. 12c,d), the Bi white nanosize precipitates were also formed in the matrix during a solid state process due to the reduced solubility of Bi in Sn by reducing the temperature. Therefore, Bi, due to the change in the solidification rate, the growth rate of eutectic phases and the reduction of the eutectic point, makes the microstructure finer.

**Figure 13:**
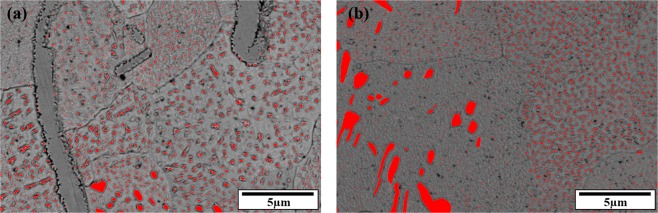
Calculating phase fraction in the solder alloys: (**a**) SAC257-2.5Bi (**b**) SAC257-5Bi.

By calculating the phase fraction in the solder alloys, which is provided in Fig. 13, it can be seen that by increasing the bismuth from 2.5 wt.% to 5 wt.%, the area fraction of bismuth precipitates increases from 4.46% to 7.54%, respectively. Bismuth, on the other hand, has inherent brittleness, and by addition of high Bi percentage to the base solder, coarse bismuth deposits are created in the microstructure, which can reduce the mechanical properties of the solder alloys. These precipitates are well visible in the microstructure of SAC257-5Bi solder alloy.

### Wettability

**Figure 14:**
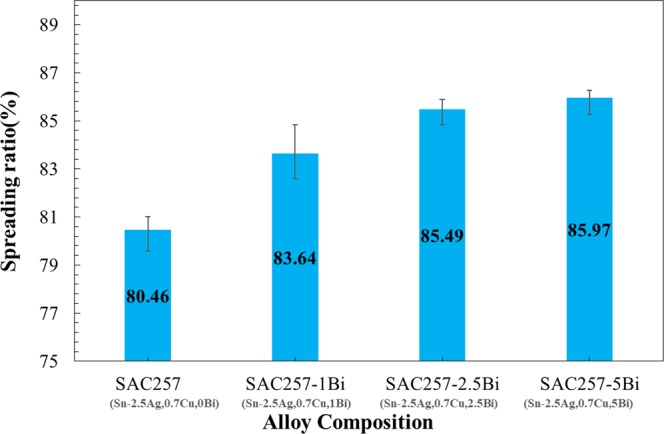
The results of spreading ratio in different solder alloys.

To achieve a sound solder joint, liquid phase should properly wet the surface of the solid base materials. This means that there should be a special reaction between the liquid solder and the solid surfaces that are supposed to be bonded. The ability of liquid solder for spreading and flow during the soldering process is one of the most important factors for the formation of a suitable bond between solder and the base material^29,30^. Therefore, for improving the properties of SAC solders with alloying elements, the alloying elements should be selected in such a way that the wettability of the solder does not decrease and even improves. The spreading ratio is one of the important factors for investigation of the wettability of alloy solders. The results of spreading ratio in different cases are shown in Fig. 14. It is observed that with the addition of bismuth to the base solder, the percentage of spreading ratio increases and ranges from 80.46% in SAC257 to 85.97 in the SAC257-5Bi solder.

The variation of the spreading ratio from 2.5 wt.% Bi to 5 wt.% Bi alloy solders is eligible, indicating that more than 2.5 wt.% Bi does not change the wettability. Generally, proper wettability occurs when there is the lowest amount of surface free energy. This means that the surface energy of the solder will be reduced by forming an interface with lower free energy. On the other hand, in order to liquid solder wets and bonds with the base material surface, the external forces (between liquid and the substrate) must overcome the internal forces (liquid interatomic forces). As the molten inner bonds become weaker, wettability can improve. In alloy solders, with the addition of bismuth and the formation of a weaker Sn-Bi bond, instead of Sn-Sn, the wettability can be improved. For this reason, the addition of bismuth to the base solder will increase the wettability^25,31^.

In terms of classical wettability model, for determining the wettability of molten solder on a solid surface, the competition between the cohesive forces (molten internal forces) and adhesive forces (the force between the atoms of the molten solder and the substrate), as well as the molten surface tension, is decisive. Therefore, in order to molten solder wets the substrate, the adhesive forces must overcome the cohesive forces of the molten solder. From the classical wetting point of view, the weaker the cohesive forces of the molten solder, the higher the wettability of the liquid phase on the solid substrate and the lower the wetting angle. As mentioned before, the melting temperature decreases in the solder alloy by adding Bi. Given this, addition of Bi weakens the internal forces (likely to form a weaker Sn-Bi bond instead of Sn-Sn) and therefore, external forces can overcome more than before. In other words, from the classical wetting perspective, by increasing the weight percentage of Bi as the melting point depressant, the spreadability of the liquid phase increases. Bi also has a lower surface tension and, by adding it to the base solder, the overall surface tension of the molten solder is reduced^31–33^.

**Figure 15:**
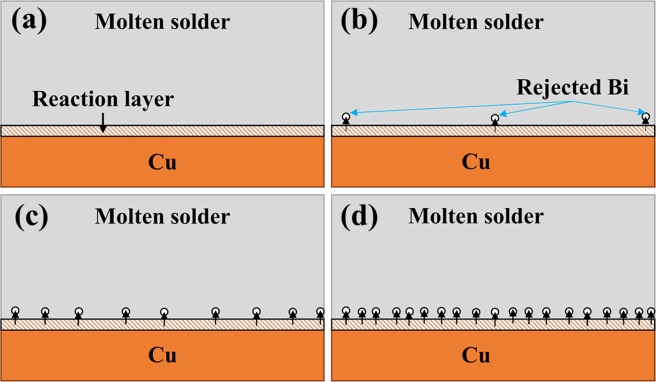
Schematic of the bismuth effect on reactivity and spreading in the interface of molten solder/Cu substrate: (**a**) SAC257/Cu (**b**) SAC257-1Bi/Cu (**c**) SAC257-2.5Bi/Cu (**d**) SAC257-5Bi-Cu.

But, in addition to the classical theory of wettability, reactive wettability should also be taken into account, because the molten solder reacts with the substrate. Thus, the reaction of the molten solder with the copper substrate also affects the wettability and spreading ratio. Studies have shown that Bi and Cu have a very poor reactivity, but Sn and Cu have a strong interaction. In general, the presence of bismuth in small concentrations, increases the reactivity between Sn and Cu, and the Sn-base solder alloys containing Bi exhibit better wettability comparing to the base solder (So not only from the classical wettability, but also from the reactive wettability point of view, the wetting should increase). But by increasing the amount of Bi in the solder alloy, the rate of increase in the wettability decreases and high concentrations of Bi, negatively affects the wettability. The reason for such a behavior can be explained based on the following schematic. Regarding Fig. 15a–d, for the reaction and the formation of equilibrium intermetallic compounds at the interface, because of the weak reactivity of Bi with Cu, Bi atoms dissolved in β-Sn will be removed from this structure due to the reduction of the local solubility. The greater the amount of Bi in the solder, the more Bi atoms accumulate at the interfacial molten phase reducing the effective reactivity of Sn and Cu^34^. As shown in the results of the spreading coefficient, the rate of increase in wettability decreases with increasing Bi weight percent (see Fig. 14).

### Tensile properties of the solders

**Figure 16:**
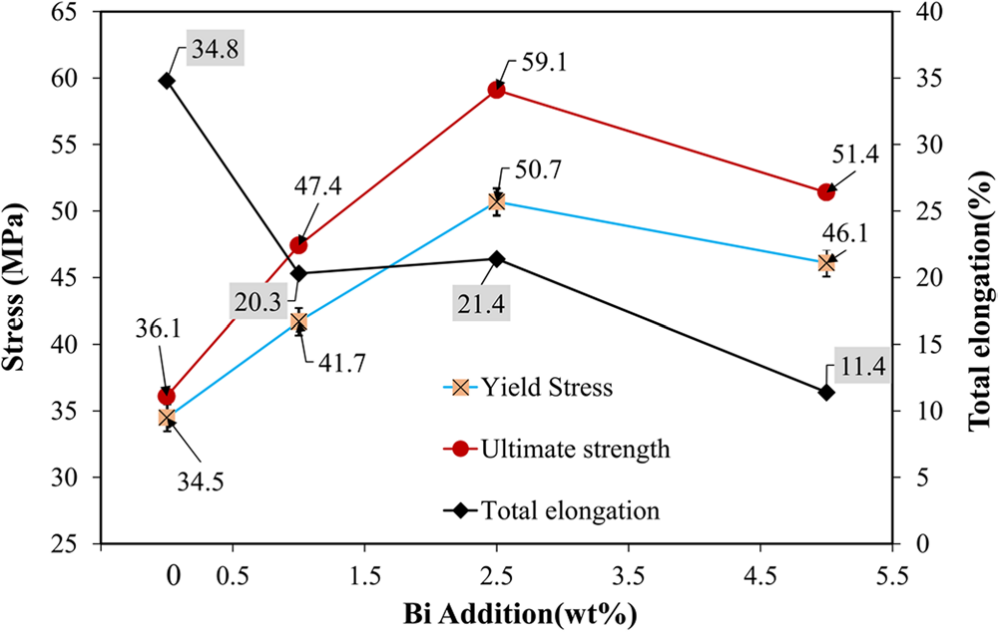
The tensile test results of the SAC257 base solder and SAC257-xBi alloy solders.

Figure 16 shows the tensile test results of the SAC257 base solder and SAC257-xBi alloy solders. According to the results, for the alloy solders with bismuth up to 2.5 wt.%, the yield stress and the tensile strength increase, and the percentage of elongation shows a gradual decreasing trend. However, in the SAC257-5Bi alloy solder, the yield and tensile strengths are reduced and the percentage of elongation is also significantly reduced. According to the SEM microscopic images (Fig. 9d), the large bismuth precipitates are visible in the microstructure of SAC257-5Bi alloy solder. Bismuth is inherently brittle, dissolves in small amounts in the Sn matrix (about 1 wt.%) and the high percentage of bismuth in the Sn alloy forms large precipitates in the matrix by solid state precipitation of Bi. Therefore, the high amount of Bi is detrimental for the mechanical properties. In low percentages of bismuth (<5 wt%), the strength can increase through the formation of a solid solution with Sn, limiting the growth of intermetallic compounds and formation of fine precipitates. In fact, these alloy solders (1 and 2.5 wt.% Bi) are the same as *in-situ* composites, while fine bismuth precipitates and IMCs are formed in the solder matrix during the cooling stage. Since these precipitates have a suitable chemical bonding with the matrix, their interaction with dislocations, slows their movement, delays the plasticity and increases the strength^35,36^.

### Joint properties

#### Microstructure of the interface

**Figure 17:**
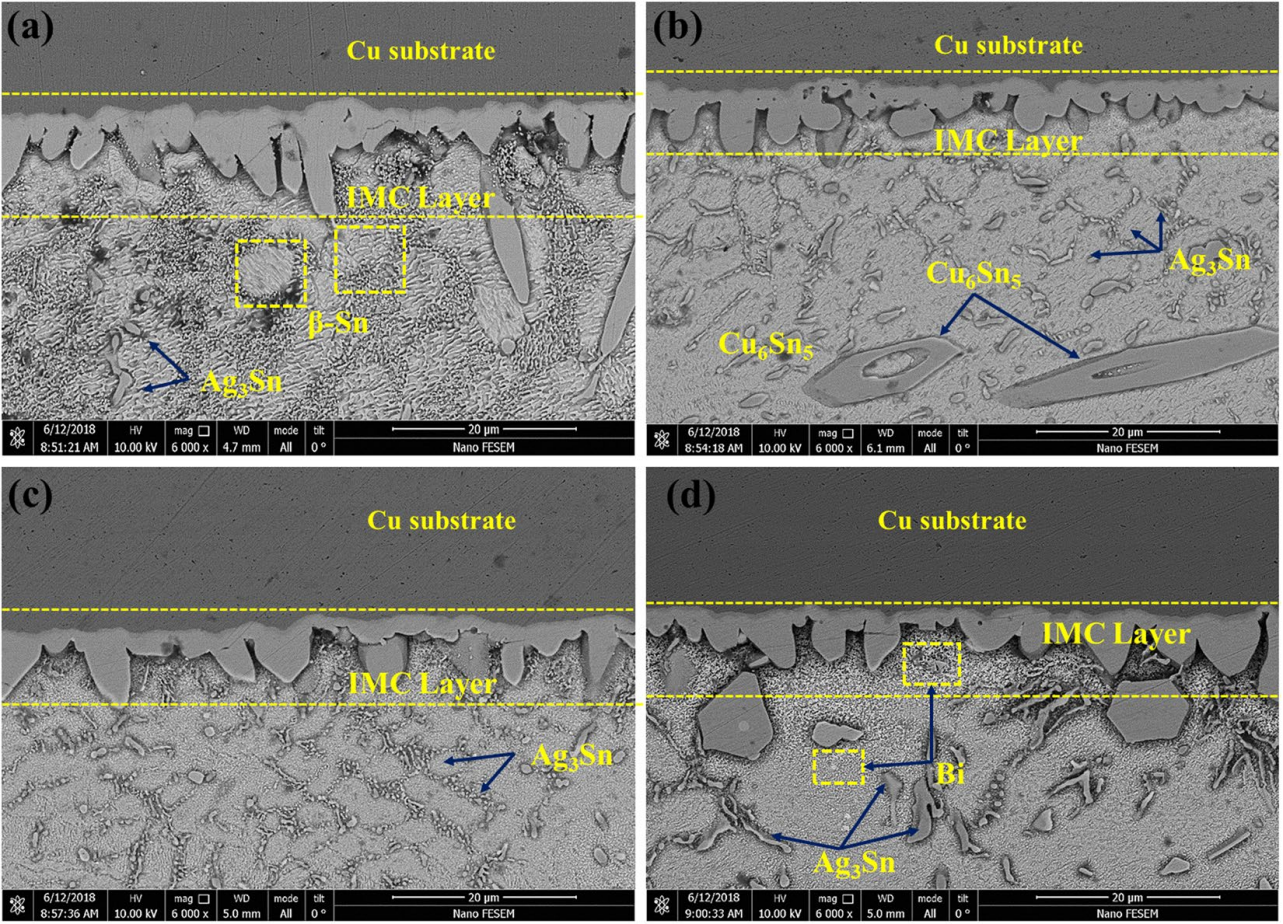
The FE-SEM back scatter electron images of the microstructure at the interface of the solder base and solder alloys bonded to the copper substrate after the soldering process: (**a**) Cu/SAC257/Cu (**b**) Cu/SAC257-1Bi/Cu (**c**) Cu/SAC257-2.5Bi/Cu (**d**) Cu/SAC257-5Bi/Cu.

Figure 17 shows the FE-SEM back scatter electron images of the microstructure at the interface of the solder base and solder alloys bonded to the copper substrate after the soldering process. It is observed that due to the reaction of Sn and Cu between the solder and copper substrate, the scallops shaped intermetallic compounds are formed at the interface. By adding Bi to the alloy solders, the morphology and thickness of the intermetallic compounds are converted from the bar/columnar type to the co-axial/round type. According to Fig. 4, for obtaining the thickness of the interface IMCs, the total area of the IMC layer was calculated, and then the thickness of the IMCs was measured by Equation (2) and is given in Table 3.

**Table 3:** The thickness of the IMCs measured by equation (2).

Joint	IMC Thickness (µm)
SAC257/Cu	4.83
SAC257-1Bi/Cu	4.16
SAC2572.5Bi/Cu	4.23
SAC257-5Bi	4.36

**Figure 18:**
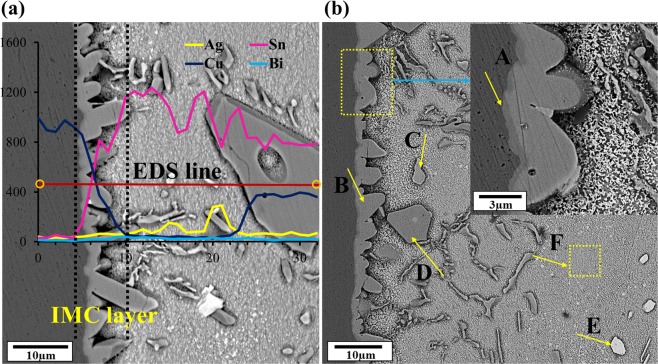
The EDS analysis to identify the interface and matrix phases in SAC257-5Bi solder alloy: (**a**) linear EDS (**b**) point EDS (**c**) table of result for point EDS.

It is observed that the thickness of the IMCs formed at the interface changes from 4.83 μm in the SAC257 solder to; 4.16, 4.23 and 4.36 µm in 1, 2.5 and 5 wt.% Bi alloy solders, respectively. It can be said that the thickness of IMCs in SAC257-xBi solders has decreased. For example, in the case of SAC257-1Bi alloy solder, the thickness is reduced by about 14% relative to the base solder. The linear and point EDS analyses are used to identify and analyze the interface and matrix phases which are shown in Fig. 18a–c. According to the semi-quantitative results and the previous research studies, the intermediate IMCs can be characterized as Cu_6_Sn_5_^37^. Due to the limited solubility of Sn and Cu in each other and after dissolution of the copper in a short period of time in liquid phase, the adjacent liquid layer on the substrate at the interface saturates with Cu atoms (dissolved substrate). According to the equilibrium phase diagram, intermetallic compounds on this layer begin to form and generate at the interface by the following reaction^38–40^:

3 $$6Cu+5Sn\to C{u}_{6}S{n}_{5}$$

In addition to the Cu_6_Sn_5_ interlayer, a very thin interlayer with a darker color compared to Cu_6_Sn_5_, is formed between the Cu_6_Sn_5_ and the substrate, which can be deduced to be Cu_3_Sn from EDS results and 3:1 ratio of Cu/Sn. This phase has a super lattice structure with long-range atomic arrangement. Formation of this thin layer is according to the following reaction^41,42^:

4 $$C{u}_{3}Sn\to 3Cu+Sn$$

In addition to the mentioned IMCs at the interface, other IMCs have been observed in the solder matrix. In the case of SAC257, Ag_3_Sn and Cu_6_Sn_5_, and in the case of SAC257-5Bi solder alloy, bismuth deposits besides Ag_3_Sn and Cu_6_Sn_5_ have also been observed (EDS Fig. 18b,c).

The relationship between the thickness of IMC layer and atomic diffusion is generally considered to follow the Fick’s law^43^.

5 $$Y={(Dt)}^{n}$$

In which Y is the thickness of the IMC layer, t is the time, D is the atomic diffusion coefficient and n is the power constant. Diffusion of Sn and Cu is the primary mechanism for controlling the thickness of IMCs. Generally, bismuth does not react with Cu, hence bismuth alone does not change the reaction layer at the interface. Li Guo-yuan and Shi Xun-qing have investigated the effect of bismuth on the growth of intermetallic compounds in SAC-xBi solders on copper substrate, and they have shown that the addition of bismuth up to 1 wt.% to the Sn-3.8Ag-0.7Cu solder increases the activation energy, and more than 1 wt.% Bi decreases the activation energy^43^. Consider the following equation (6):

6 $$D={D}_{0}\,\exp (\frac{-Q}{KT})$$

**Figure 19:**
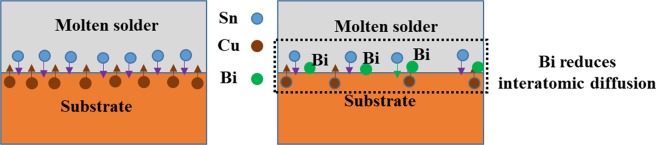
The proposed mechanism for the effect of Bi on the thickness of the intermetallic compound at the interface.

Where D is the interdiffusion coefficient; D_o_ is the pre-exponential constant of interdiffusion coefficient; Q is the activation energy, k is the Boltzmann constant and T is the absolute temperature. By increasing the activation energy, the interdiffusion coefficient decreases, and vice versa. The reason for the reduction in the thickness of the interface IMCs in the SAC257-1Bi solder can be attributed to the increase in activation energy of the solder, which reduces the interdiffusion coefficient and therefore the reaction rate between molten Sn and the substrate. On the other hand, the copper solubility in the molten solder will occur at a lower rate, and the thickness of the intermetallic compound decreases at the interface (schematic Fig. 19). By increasing the bismuth fraction in the SAC solders, the activation energy decreases and with the increase in the diffusion rate at the interface, the thickness of the IMCs increases. Feng *et al*.^34^ used the molecular orbital method and found that the presence of Bi and Ag increases the orbital interaction between Sn and Cu for 1.68% and 0.67%, respectively. In fact, Bi and Ag act as catalyst, although they do not participate in the reaction themselves, increase the reaction rate of Sn with Cu. This increase in reaction rates seems to occur at high weight percentages of bismuth^34^. In this study, the weight percentage of Ag in the SAC solder was reduced compared to its eutectic composition, which could be effective in reducing the interface reaction rate.

**Figure 20:**
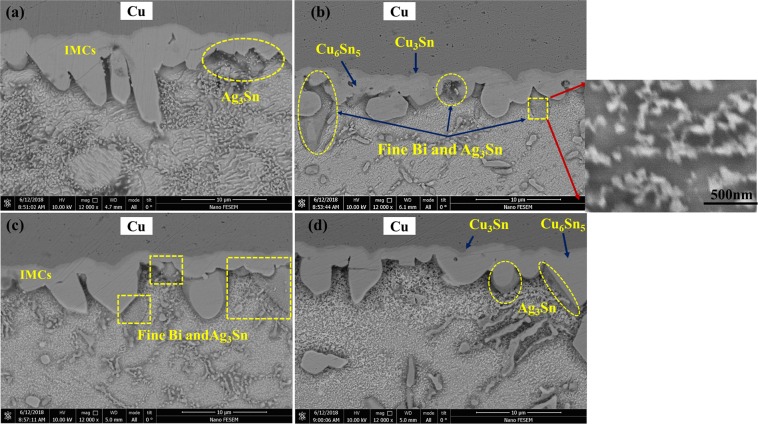
The interfacial IMCs showing bright tiny deposits near the interface and on the IMCs with nanometer dimensions: (**a**) Cu/SAC257/Cu (**b**) Cu/SAC257-1Bi/Cu (**c**) Cu/SAC257-2.5Bi/Cu (**d**) Cu/SAC257-5Bi/Cu.

On the other hand, as mentioned, bismuth increases the solidification rate by reducing the melting temperature and degree of undercooling, and therefore limits the growth of IMCs. By reducing the melting temperature of SAC257 base solder by adding Bi, the soldering process can be carried out at a temperature below the SAC257 melting point, so that the solder with a lower melting temperature is placed on the copper substrate during soldering, which can slow down the diffusion of Sn to the substrate and, as a result, the solubility of the copper substrate inside the solder, which reduces the thickness of the IMC layer. According to Fig. 20, a closer look at the interfacial IMCs shows that bright tiny deposits have been deposited near the interface and on the IMCs with nanometer dimensions. The amount of precipitates increases with the addition of bismuth, and their dimensions become smaller. According to previous studies^44,45^, these precipitates are Ag_3_Sn particles that have been produced by reducing the degree of undercooling. The precipitation of Ag_3_Sn particles on IMCs reduces the surface energy and can prevent the growth of intermetallic compounds during electronic equipment service.

#### Tensile-shear properties of the joint

**Figure 21:**
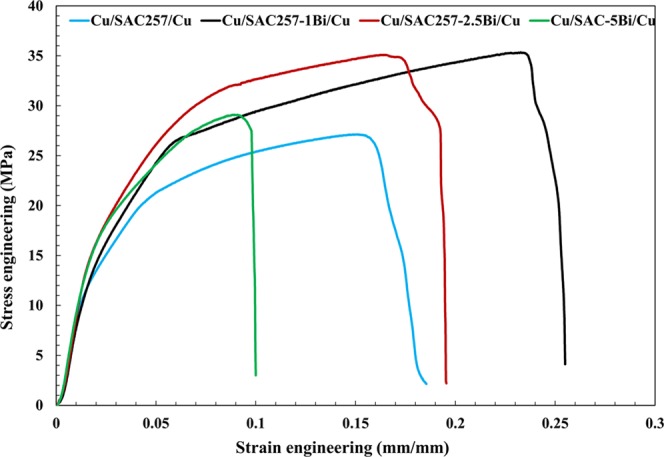
The stress-strain curves of solder joints obtained from the tensile-shear test.

To investigate the mechanical properties of solder joints, the tensile-shear test is one of the most suitable tests. In this test, the interfacial region is effectively subjected to stress and the relationship between mechanical properties and its microstructure is properly evaluated^19^. In Fig. 21 and Table 4, the average mechanical properties of solder joints in the tensile-shear test are represented. As it is obvious, the tensile strength of the joints initially shows an ascending trend with the increase in weight percentage of Bi and then shows a descending trend. But in general, the tensile-shear strength of all bismuth containing solders is higher than the base solder.

**Table 4:** The average mechanical properties of solder joints in the tensile-shear test.

Solder alloy	Yield strength (MPa)	Ultimate strength (MPa)	Elongation (%El)	Failure location
SAC257	21.92 ± 2	27.13 ± 2	18.51 ± 3	Solder-IMC
SAC257-1Bi	26.49 ± 2.5	35.34 ± 1	25.49 ± 2	Solder
SAC257-2.5Bi	31.06 ± 1	35.10 ± 1	19.54 ± 3	Solder-IMC
SAC257-5Bi	26.88 ± 1.5	29.1 ± 3	10 ± 1	IMC

The SAC257-1Bi alloy solder joint has the maximum strength and elongation, which are 30% and 38% higher than that of SAC257 base solder joint, respectively. Thickness of the IMCs at the interface is represented in Table 3. The interfacial IMCs in the solder joints play a major role in mechanical properties, so that by decreasing the thickness of the IMCs by adding bismuth, strength and ductility increase simultaneously. However, as mentioned in the previous sections, bismuth is inherently brittle and with the formation of large bismuth precipitates in the matrix, ductility decreases. In addition, the interfacial IMCs reduce the strength and ductility of the joint which is valid for SAC257-5Bi joint. In the case of SAC257-1Bi joint, Bi increases the strength and elongation of the joint by means of solid solution with Sn, reducing the size of the Ag_3_Sn intermetallic compound and the thickness of the IMCs at the interface. In the case of SAC257-2.5Bi joint, formation of Bi precipitates in the matrix and decreasing the size of the IMCs are responsible for the higher strength. But in comparison with the case of 1wt.% Bi, bismuth precipitates are also present in the matrix, which prevent the movement of dislocations, delay plasticity and decrease ductility^25^.

#### Fracture mechanism of the joint

**Figure 22:**
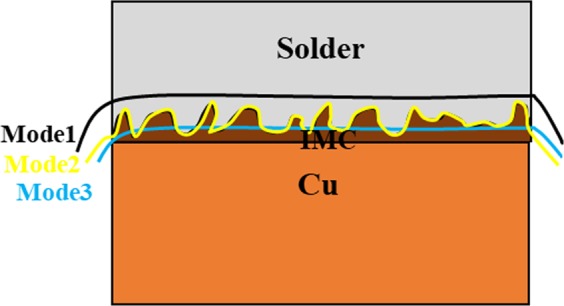
The three general modes (mechanisms) of failure in solder joints.

**Figure 23:**
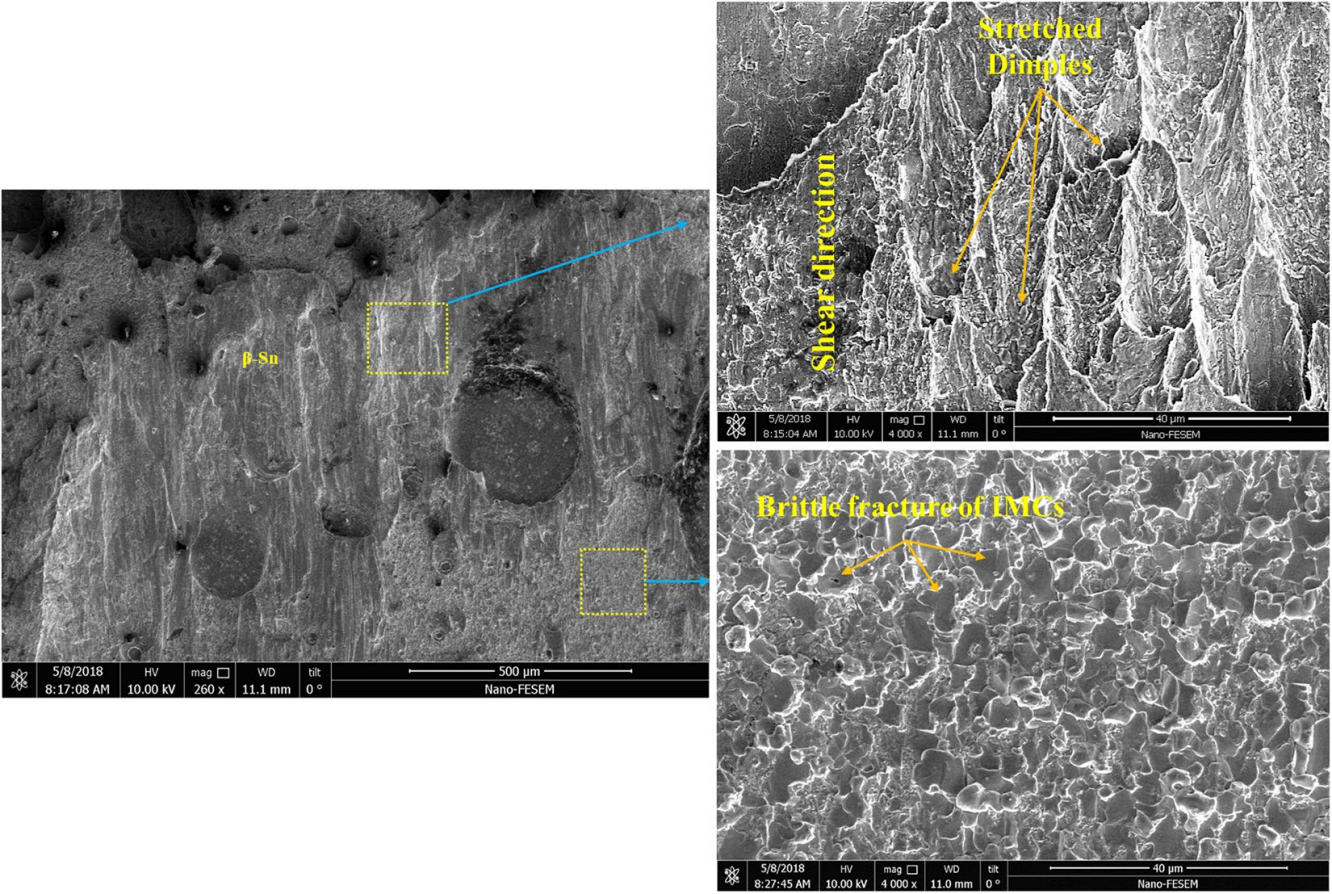
The fractured surfaces of the SAC257/Cu.

**Figure 24:**
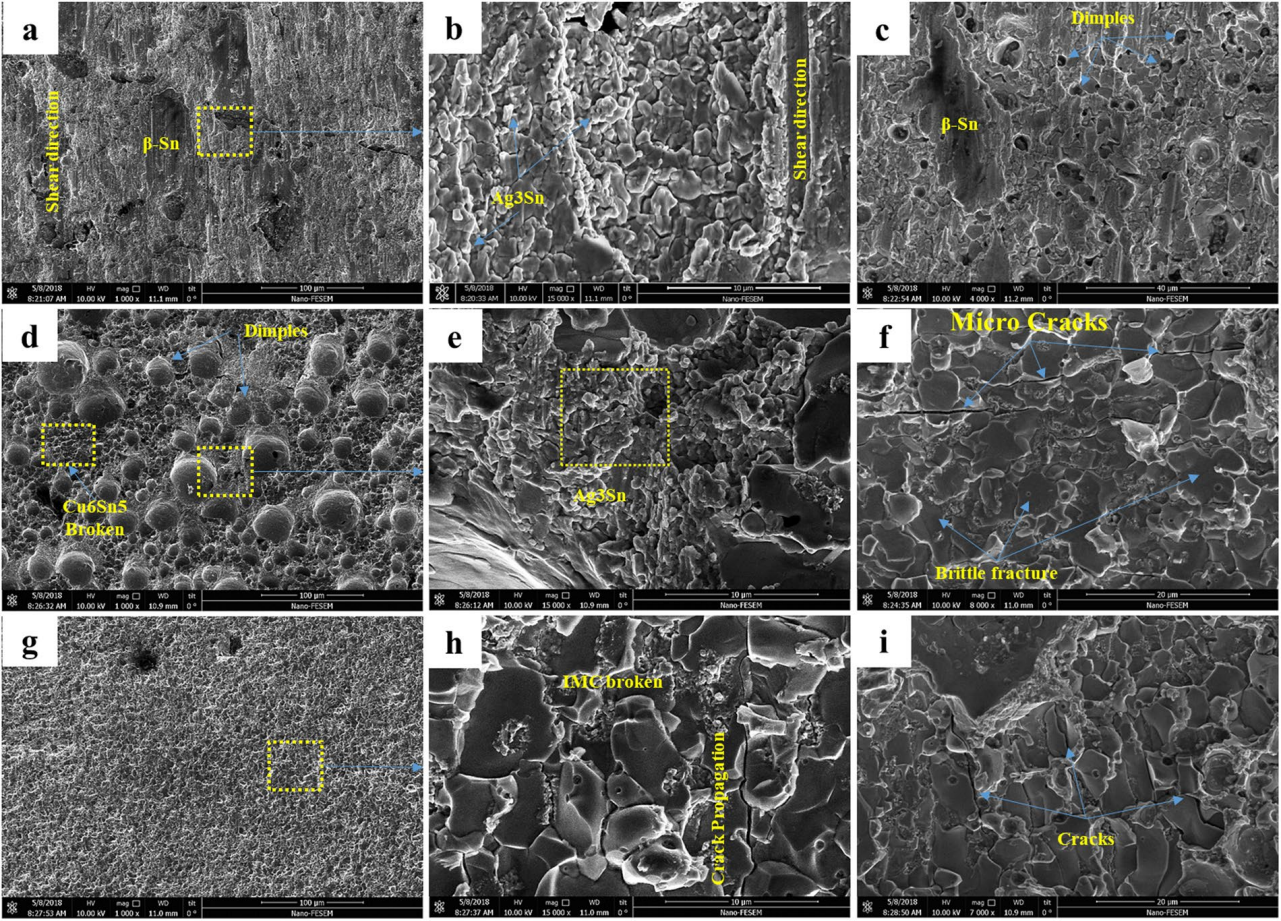
The fractured surfaces of SAC257-xBi alloy solders: (**a**–**c**) SAC257-1Bi/Cu (**d**–**f**) SAC257-2.5Bi/Cu (**g**–**i**) SAC257-5Bi/Cu.

**Figure 25:**
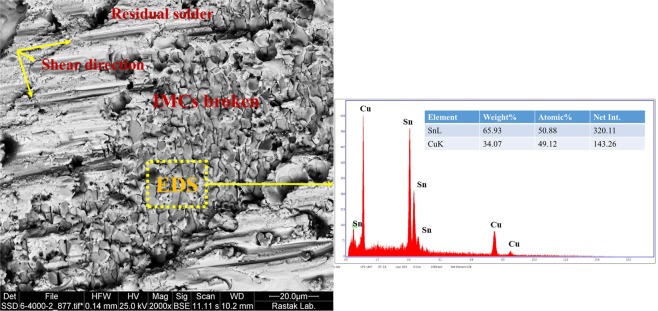
The BSE images of the fracture surface in the case of SAC257-2.5Bi alloy solder.

In order to investigate the fracture modes, the fractured surfaces after tensile-shear test were studied and the results are presented in Figs 22–25. Generally, when shear force is applied to the solder joints, more stress is concentrated at the interfacial region. Since, the interfacial IMCs are brittle, the tendency for interfacial fracture (IF) is very high, that is why the thickness of the IMCs at the interface and the strength of the solder matrix are very influential in fracture mode. According to Fig. 22, three general modes of failure in solder joints can occur^46^: (I) represents a soft fracture that occurs from the bulk of the solder. This state occurs when the thickness of the IMC and the strength of the solder matrix are low. Due to the higher strength of IMCs, it is broken down from the solder matrix, (II) this mode occurs when the crack propagates in the boundary of the IMCs and creates a detachment. This mode of failure is more common in tensile mode of tensile-shear test. The smaller the IMCs, the longer the crack propagation and the more the energy absorption. In this mode, mixed fracture (ductile-brittle) occurs, (III) in this mode, which indicates a brittle fracture, the cracks develop inside the IMCs, and the separation of the solder from substrate takes place within the IMCs. Therefore, at the fracture surface, broken IMCs are visible. Mode (III) occurs in a situation where the thickness of the IMCs or the strength of the solder matrix is high^46–48^. However, these three scenarios may occur at the same time in a joint due to microstructure changes and variation in the thickness of the IMCs across the interface. According to the fractured surfaces of the SAC257/Cu in Fig. 23, fracture in a part of the surface is ductile and the stretched dimples under the shear force are visible and in the remaining part is brittle (lack of dimples). By considering the thickness of the IMCs at the interface and the shear strength, failure behavior is justifiable. In Fig. 24a–i, the fractured surfaces of SAC257-xBi alloy solders have been shown. As seen in the SAC257-1Bi solder, it is generally broken from the solder bulk, and the failure dimples and shear directions are visible at the surface which indicate a ductile fracture. The low thickness of the interfacial IMCs and the Bi solubility in Sn are the main factors that cause a ductile fracture. According to the fractured surfaces of SAC257-2.5Bi solder, there are both symptoms of ductile and brittle fracture, which can be said to be the combination of all three failure modes presented in Fig. 22. Detachment of the IMCs, presence of dimples (Fig. 24e) and also propagation of the crack inside the IMCs (Fig. 24f) all indicate that there is a mixed mode fracture (ductile-brittle) in this case. The SAC257-5Bi alloy solder surfaces are completely flat and fractured IMCs are visible at the surfaces, indicating the third mode of failure (see Fig. 24g–i). Presence of thick interfacial IMCs and the bismuth precipitates in the microstructure make the third mode of failure favorable.

Figure 25 shows the BSE images of the fracture surface in the case of SAC257-2.5Bi alloy solder. According to this figure, ductile-brittle failure is obvious and the EDS from the selected point represents the Cu_6_Sn_5_ phase at the surface that means a part of failure is brittle.

## Conclusion

In this study, alloying of Pb-free Sn-2.5Ag-0.7Cu solder (containing low Ag content) with Bi was investigated. Bulk and joint (Cu/SAC-xBi/Cu) properties of the solder alloys were studied and the main achievements are summarized below:

1. It was observed that the thermal properties of the SAC257 improve with adding Bi, and the eutectic temperature (melting temperature) was reduced 2 °C per weight percent of Bi element. Degree of undercooling, liquidus and solidus temperatures were decreased as well, while pasty range did not experience significant changes in 1, 2.5 wt.%Bi alloy solders.
2. By adding Bi to the base solder, tensile strength increased by solid solution strengthening mechanism and the formation of fine bismuth precipitates in the matrix. But in high Bi contents (5 wt.%Bi), the tensile strength and elongation percentage decreased due to formation of course Bi-rich precipitates. By considering the spreading ratio in base and alloy solders, Bi has enhanced the wettability of the solder alloys.
3. Adding bismuth alters the morphology and thickness of Cu_6_Sn_5_ interfacial IMCs in such a way that IMCs thickness shows a descending trend at first, followed by an ascending trend which is generally thinner than the base solder interfacial IMCs. In the case of SAC257-1Bi joint, the interfacial IMCs is minimum which shows a 14% reduction in the thickness comparing to base solder.
4. By comparing the fracture surfaces of base solder (SAC257) and alloy solders (SAC257-Bi), it was observed that the fracture mechanism changes from IMC control (low reliability) in base solder joint to solder control (ductile fracture) in SAC257-1Bi joint, which is more reliable.
5. Bi white nanosize precipitates were also formed in the matrix during a solid state process due to the reduced solubility of Bi in Sn by reducing the temperature. Therefore, Bi, due to the change in the solidification rate, the growth rate of eutectic phases and the reduction of the eutectic point, makes the microstructure finer.
6. Addition of Bi weakens the internal forces (likely to form a weaker Sn-Bi bond instead of Sn-Sn) and therefore, external forces can overcome more than before. In other words, from the classical wetting perspective, by increasing the weight percentage of Bi as the melting point depressant, the spreadability of the liquid phase increases. Bi also has a lower surface tension and, by adding it to the base solder, the overall surface tension of the molten solder is reduced.
